# Microalgae-Based Green Bio-Manufacturing—How Far From Us

**DOI:** 10.3389/fmicb.2022.832097

**Published:** 2022-02-17

**Authors:** Hui Chen, Qiang Wang

**Affiliations:** ^1^State Key Laboratory of Crop Stress Adaptation and Improvement, School of Life Sciences, Henan University, Kaifeng, China; ^2^Academy for Advanced Interdisciplinary Studies, Henan University, Kaifeng, China

**Keywords:** microalgae, green bio-manufacturing, synthetic biology, gene/genome editing technology, metabolic regulation, biomass, photosynthetic efficiency

## Introduction

**Figure 1:**
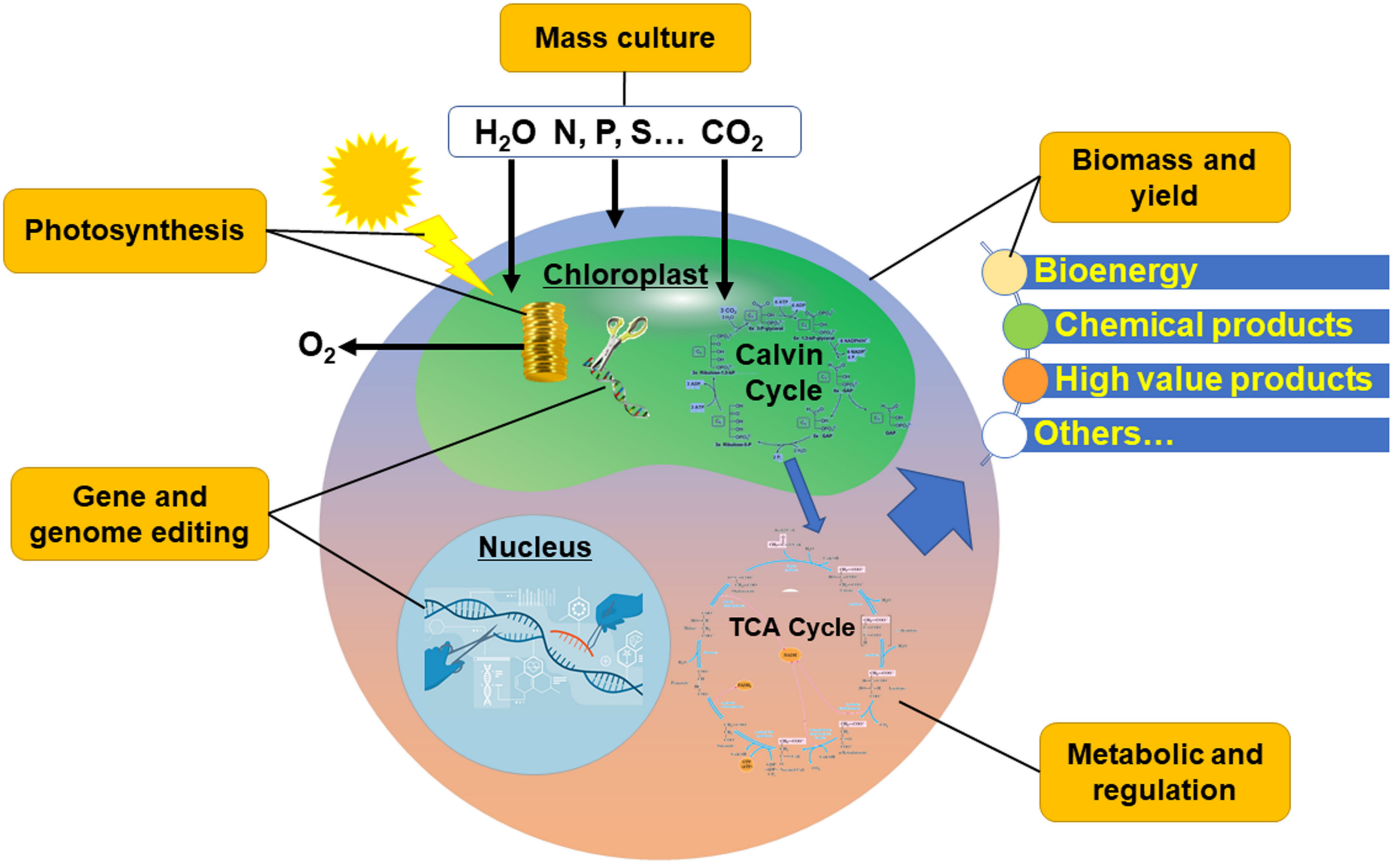
The optimized key points for microalgae-based green bio-manufacturing.

In the early twenty-first century, with the integration of engineering strategies with modern biology, systems science and synthetic science, “Synthetic Biology” has been proposed (Chen et al., 2019). Further, a new bio-manufacturing industry, which can provide industrial commodities based on large-scale material processing and transformation with biological function, was generated. At present, the production and manufacture of fuels and bulk chemical products mainly rely on petrochemical refining (Speight, 2021), which is facing the challenges of high risk of production safety, great pressure of environmental protection, and contradiction between supply and demand of oil and gas resources. Bio-manufacturing is a new manufacturing mode that breaks away from the route of petrochemical industry, and has the typical characteristics of low carbon, recyclable, green, and clean. Microalgae can use solar energy to fix CO_2_ and convert C1 compound into organic matter, which can further generate a variety of metabolites through a variety of metabolic pathways and then produce a variety of biofuels and fine chemicals through genetic engineering modification (Qin et al., 2012; Maheswari et al., 2018; Chen and Wang, 2021). Therefore, microalgae have attracted much attention in recent years as “green cell factories” (Machado and Atsumi, 2012). Compared with heterotrophic chassis cells, microalgae-based synthetic biology and bio-manufacturing also play a role in carbon sequestration and emission reduction (Chen and Wang, 2020). Developing microalgae-based green bio-manufacturing industry is expected to solve the problems of current energy crisis and the unsustainable production of chemical products, and alleviate the greenhouse effect at the same time. However, in general, microalgae have the disadvantages of low product content and high cost in cell culture and collection when producing target products. It is necessary to sort out the key problems one by one from the upstream cell factory construction to the downstream microalgal culture engineering (Figure 1).

## The Systematic Establishment of Gene/Genome Editing Technology is the Prerequisite

The premise of constructing industrial microalgae cell factories is the establishment of accurate, efficient, and stable genetic transformation and gene/genome editing system. The genetic transformation methods, including natural transformation (homologous recombination), gene gun, glass bead, electric transformation and agrobacterium mediated transformation, have been only successfully applied in a small number of model algae strains (Feng et al., 2009; Kilian et al., 2011; Qin et al., 2012; Rathod et al., 2013; Run et al., 2016; Zhao et al., 2020). It is an urgent need to develop efficient, stable and widely adaptable heterologous gene transformation technology. In recent years, some gene/genome editing technologies such as ZFN, TALEN, CRISPR/Cas, and Golden Gate (CyanoGate) have been continuously tried to be applied in microalgae genome editing, and have already been applied in *Chlamydomonas rheiniscens, Phaeodactylum tricornutum, Nannochloropsis*, and some model cyanobacteria (Sizova et al., 2013; Daboussi et al., 2014; Jiang et al., 2014; Nagel, 2019; Vasudevan et al., 2019; Ryu et al., 2021). However, these techniques have some defects, such as cytotoxicity, high off-target rate, unstable inheritance of transformers, difficulty in large fragment operation, and high interspecific specificity. Therefore, how to improve the accuracy and universal applicability of gene/genome editing technology, reduce the off-target effect, and improve the stability of transformants are the basis of microalgae genetic modification. In addition, when constructing algal strains with specific functions using multiple rounds of gene editing, traceless editing and large fragment gene manipulation techniques also need to be paid attention to.

## The Deep Understanding of Metabolic Flow and Its Regulation is the Basis

Microalgae synthesize carbohydrates by fixing CO_2_ through photosynthesis, and further generate various organic compounds through various metabolic pathways. At the same time, different types of nitrogen-containing compounds absorbed during the growth of microalgae can be used to synthesize proteins and other nitrogen-containing substances, and convert nitrogen into biomass (Chen et al., 2017; Calzadilla et al., 2019; Chen and Wang, 2020, 2021). However, while the diversity of microalgal metabolic pathways and their regulatory networks provides more opportunities for bio-manufacturing research, they are also “incredibly unpredictable” compared to other biological processes due to their complex influence variables. Up to now, the synthetic pathways of many algal metabolites are only speculated, and there is still a lack of direct experimental evidence on the specific synthetic pathways, regulatory processes and metabolic flows of C and N elements. Therefore, focusing on the key metabolic pathways including photosynthesis, Calvin cycle, tricarboxylic acid cycle and glycolysis, and clarifying the flow, distribution, redistribution, and related regulation processes of metabolic flow in microalgae under different environmental conditions are the basis for efficient synthesis of algal compounds and development of microalgae-based green bio-manufacturing. In addition, the feasibility and economy of metabolic pathway integration strategies or enzyme modification strategies in the construction of different types of chassis cells can also be compared through the method of life cycle assessment (Davis et al., 2021), which can provide guidance for the feasibility and economy of producing different types of chemicals in microalgae cell factories.

## Biomass and Yield Determine Feasibility

Compared with other heterotrophic expression vectors, microalgae have the advantage of directly converting C1 compound into organic matter without using expensive substrates. However, in order to truly realize the industrialization of microalgae-based green bio-manufacturing and meet the needs of the vast number of consumers for biological products, the relatively low biomass of microalgae would be a fatal disadvantage. Therefore, the availability of biomass and yield matching with industrial production determines the real feasibility of microalgae-based green bio-manufacturing. In order to solve the bottleneck of microalgal biomass and yield, future work can focus on: (1) The screening of high-biomass, fast-growing microalgal strains, for example, *Chlamydomonas* and cyanobacteria. (2) The other direction is to optimize the microalgal culture procedure for improving the biomass yield of microalgae. For example, the mixotrophic culture method can obtain higher biomass and yield than the autotrophic or even heterotrophic culture mode (Zhan et al., 2017). (3) The input of industrial waste as nutrient element in the mixotrophic cultivation mode can reduce production cost and bring considerable environmental benefits (Chen et al., 2016; Chen and Wang, 2020). (4) Combining with the feedback process of actual production, the establishment of efficient and low-cost cultivation and process optimization control strategy can also effectively improve microalgal biomass yield and reduce production cost.

## The Improvement of Photosynthetic Efficiency is the Fundamental Problem

Photosynthesis, the most important and largest biochemical process on earth, is the fundamental source of oxygen in the earth's atmosphere and primary productivity of the biosphere (McCourt, 2021). With long-term evolution, microalgae have been able to harness solar energy in a variety of efficient ways to improve environmental adaptability (Trentacoste et al., 2015). While the light conversion efficiency of microalgae can reach 8–10%, it's only 1–2% in actual culture. Thus, improving the photosynthesis efficiency is the fundamental problem to be solved in microalgae-based green bio-manufacturing in the future. Traditional ways to improve photosynthetic efficiency in algal culture focused on reactor system, thus a direction that needs to be paid attention to in the future is the innovation and optimization of the reactor system for improving light energy utilization efficiency. With the development of synthetic biology technology, more and more attention has been paid to the modification of algal chassis cells themselves, and the specific optimization process can be started from the light energy capture and the light energy conversion. In terms of light energy capture, introducing exogenous chlorophyll genes (Chl d and Chl f) that can absorb additional spectrum into microalgae chassis cells is likely to obtain optimized chassis cells strains with higher photosynthetic capacity. Optimization of light energy conversion focuses on improving carbon fixation mechanisms and optimizing the Calvin cycle. The most direct strategy is to effectively improve the carbon sequestration capacity of chassis cells by identifying and overexpressing specific inorganic carbon transporters and Rubisco or replacing them with more efficient enzymes.

## Microalgal Culture Optimization is the Key Link for Future Industrialization

Despite the great potential of the diverse microalgal species available and numerous advances on microalgae biotechnology, only a few microalgae species are currently produced on large-scale or commercial scale. The development of green bio-manufacturing is hindered by some limiting factors in microalgal cultivation, especially in large-scale cultivation. In particular, algal cultivation is limited by the cost of production balanced against the value of the end-product, and the high cost of the resources required for microalgal cultivation, including water, inorganic nutrients (mainly nitrogen and phosphate), and CO_2_, hinders the commercialization of microalgal production (Chen et al., 2020). It is necessary to explore ways to reduce the overall production cost of microalgae-based green bio-manufacturing by ensuring the supply chain and sustainability of nutrients required for microalgal cultivation in an economic and reasonable mode. For economic and environmental considerations, the culture medium can be recycled to reduce the cost of large volume of water consumption in microalgal cultivation. However, considering the enriched organic matter and other substances in harvested medium inhibits the subsequent cultivation of algae, it should be paid attention to the development of appropriate processes to treat the medium prior to medium replenishment and reuse. For example, UV-based photolysis has been applied in the degradation of organic matter in the culture medium and converted the growth-inhibiting fraction of organic matter in recycled medium into a nutrient source for algal growth (Wang et al., 2018). In addition, the components in some wastes, such as flue gas (mainly NOx and CO_2_), wastewater (mainly C, N and P) and waste residue (mainly Mg, K, Ca, P, Fe, etc.), have been confirmed to be used by microalgae as nutrient elements for microalgal culture, which can reduce the cost of microalgal culture while obtaining environmental benefits (Chen et al., 2016, 2018; Wang et al., 2019; Tan et al., 2020). The development of technology processes to efficiently utilize nutrients in wastes for the microalgal culture is a future direction that needs attention. In order to avoid the high cost of extraction and purification caused by physical and chemical properties and complex metabolites of different algae species in the later refining process of targeted high-value products, effective maintenance of mono algal cultures needs to be focused on, and development of closed photo-bioreactors with better performance will be an important solution. In addition to the improvement of culture technology to avoid contamination, more attention should be paid to improve the design of bioreactors. For example, thin-film flat plate photo-bioreactors and biofilm photo-bioreactors offered many advantages over conventional cultures, such as lower water use, higher light penetration efficiency easier harvesting, less contamination, and easier scale-up (Sun et al., 2019; Wu et al., 2019).

## Discussion

Microalgae are the oldest and most important primary producers in the world and important model system for studying photosynthesis and secondary metabolism. The research on the basic theory and application of green bio-manufacturing based on microalgal synthetic biology is of great practical significance to solve the environmental, resource, energy and food problems faced by human beings and realize the sustainable development of society. At present, pharmaceutical raw materials, feed pigments, functional foods and dietary supplements based on microalgae bioactive products are expensive and in great demand in the domestic and foreign markets, which has great commercial prospect and economic value. As the new research area and industry direction, microalgae-based green bio-manufacturing adds new genes and inserts functional biological systems to the chassis cell platform, so that it breaks through the regulation and efficiency of natural biosynthesis limits and let the cell make the material that human needs, which has important practical significance. However, as the current status mentioned above (Table 1), microalgae-based green bio-manufacturing is still in its infancy, and most of the research is still at the preliminary exploration level, so there is still a long way to go before it is really applied in practice. Finally, as summary of future solutions in Table 1, with the establishment of a perfect gene/genome editing platform, a deeper understanding of microalgae metabolic pathway, further improvement and optimization of photosynthetic efficiency of algae chassis cell, further optimization of microalgal culture process and equipment, and further improvement of biomass and yield, it will eventually be possible to engineer microalgal cell factories for producing various chemical products based on synthetic biology techniques.

**Table 1:** Current status and future solutions of key issues related to microalgae-based green bio-manufacturing.

**Key issues**	**Current status**	**Ideal state and solutions–Future directions**
Gene and genome editing	The current genetic transformation methods and gene/genome editing technologies have been only successfully applied in a small number of model algae strains, and some defects have been existed.	Develop efficient, stable and widely adaptable heterologous gene transformation technology; Improve the accuracy and universal applicability of gene/genome editing technology; Traceless editing and large fragment gene manipulation techniques need to be paid attention to.
Metabolic and regulation	Metabolic pathways and their regulatory networks are “incredibly unpredictable” compared to other biological processes; the synthetic pathways of many algal metabolites are speculated, lacking of direct experimental evidence.	Focus on the key metabolic pathways, and clarify the metabolic flow and related regulation processes under different environmental conditions; develop life cycle assessment for assessment of the feasibility and economy of producing different types of chemicals in microalgae cell factories.
Biomass and yield	The availability of biomass and yield didn't match with industrial production.	Screen high-biomass, fast-growing microalgal strains; optimize the microalgal culture procedure for improving the biomass yield; develop mixotrophic cultivation mode with industrial waste as nutrient element; establish efficient and low-cost cultivation and process optimization control strategy.
Photosynthesis	Light conversion efficiency of microalgae in actual culture is much lower than its theoretical value.	Innovate and optimize reactor system for improving light energy utilization efficiency; modify algal chassis cells by optimizing light energy capture and conversion.
Mass culture	Only a few species are currently produced on large-scale or commercial scale; algal cultivation is limited by the cost of production balanced against the value of the end-product	Develop appropriate processes to be harmless of the recycled medium for reuse; develop technology processes to efficiently utilize nutrients in wastes for the microalgal culture; develop closed photo-bioreactors and improve the bioreactors design for effective maintenance of mono algal cultures.

## Author Contributions

HC and QW conceptualized the idea for manuscript. HC drafted the manuscript. QW evaluated the manuscript and improved the content. All authors contributed to the article and approved the submitted version.

## Funding

This work was supported jointly by the National Key R&D Program of China (2021YFA0909600), the National Natural Science Foundation of China (31870041, 31770128, and 91851103), the Natural Science Foundation of Henan Province (212300410024), the Program for Innovative Research Team (in Science and Technology) in University of Henan Province (22IRTSTHN024), and the 111 Project (#D16014).

## Conflict of Interest

The authors declare that the research was conducted in the absence of any commercial or financial relationships that could be construed as a potential conflict of interest.

## Publisher's Note

All claims expressed in this article are solely those of the authors and do not necessarily represent those of their affiliated organizations, or those of the publisher, the editors and the reviewers. Any product that may be evaluated in this article, or claim that may be made by its manufacturer, is not guaranteed or endorsed by the publisher.
